# Tracheotomy

**Published:** 1874-06

**Authors:** 


Tracheotomy.—*Dr. Sayre* exhibited a string of beads, one of which he had taken from the trachea of a child seven years of age, living in New Jersey. On the 11th of February he removed the bead, which had been swallowed four days previously. She had a continuous, anxious cough, and complained of a great deal of pain under the middle of the sternum. Whenever she coughed the bead would pass up and down the trachea, and you could hear it as it struck up against the glottis. She was relieved by tracheotomy, which was performed by making a free incision in the median line and severing the tracheal rings. She coughed out the bead, gave one inspiration, and died. The incision was opened thoroughly, and artificial respiration was established, being kept up thirteen and a half minutes before any voluntary respiratory movements on the part of the patient were observed. Alcohol was injected into the rectum, and the galvanic current into the phrenic nerve. It was nearly half an hour afterward that she finally gave a cough, and a large piece of inspissated mucus appeared in the opening of the larynx, which had formed an overcoat to the bead, which was immediately seized and pulled out. I suppose the overcoat fell off from the bead and plugged up the bronchus, which was the cause of the sudden collapse. She immediately rallied, and then had no further trouble.

The point of interest is the fact of respiration being established while the tracheal incision was open; and it is a question whether we might not perform tracheotomy with advantage in cases of drowning and like accidents.

